# In Situ Anchoring Anion‐Rich and Multi‐Cavity NiS_2_ Nanoparticles on NCNTs for Advanced Magnesium‐Ion Batteries

**DOI:** 10.1002/advs.202200067

**Published:** 2022-04-24

**Authors:** Zisen Ye, Ping Li, Wutao Wei, Chao Huang, Liwei Mi, Jinglai Zhang, Jiujun Zhang

**Affiliations:** ^1^ Henan Key Laboratory of Functional Salt Materials Center for Advanced Materials Research Zhongyuan University of Technology Zhengzhou 450007 China; ^2^ Institute for Sustainable Energy College of Sciences Shanghai University Shanghai 200444 China; ^3^ Institute of Upconversion Nanoscale Materials College of Chemistry and Chemical Engineering Henan University Kaifeng Henan 475004 China

**Keywords:** anion‐rich and multi‐cavity, in situ anchoring, mechanism research, NiS_2_ nanoparticles, the storage of Mg^2+^

## Abstract

Magnesium (Mg)‐ion batteries with low cost and good safety characteristics has attracted a great deal of attention recently. However, the high polarity and the slow diffusion of Mg^2+^ in the cathode material limit the development of practical Mg cathode materials. In this paper, an anion‐rich electrode material, NiS_2_, and its composite with Ni‐based carbon nanotubes (NiS_2_/NCNTs) are explored as the cathode materials for Mg‐ion batteries. These NiS_2_/NCNTs with excellent Mg^2+^ storage property is synthesized by a simple in situ growth of NiS_2_ nanoparticles on NCNTs. NiS_2_ with both a large regular cavity structure and abundant sulfur‐sulfur (S—S) bonds with high electronegativity can provide a large number of active sites and unobstructed transport paths for the insertion–disinsertion of Mg^2+^. With the aid of 3D NCNTs skeleton as the transport channel of the electron, the NiS_2_/NCNTs exhibit a high capacity of 244.5 mAh g^−1^ at 50 mA g^−1^ and an outstanding rate performance (94.7 mAh g^−1^ at 1000 mA g^−1^). It achieves capacitance retention of 58% after 2000 cycles at 200 mA g^−1^. Through theoretical density functional theory (DFT) calculations and a series of systematic ex situ characterizations, the magnesiation/demagnesiation mechanisms of NiS_2_ and NiS_2_/NCNTs and are elucidated for fundamental understanding.

## Graphical abstract

NiS_2_/NCNTs fabricated by the in situ anchoring of NiS_2_ on the surface of Ni‐based CNTs integrates the advantages of the strongly electronegative S—S bonds and the large regular 1D cavities of NiS_2_ and the 3D conductive skeleton of NCNTs to achieve excellent Mg^2+^ storage properties.

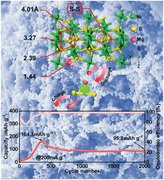

## 1 Introduction

Electrochemical energy devices including batteries and supercapacitors water electrolyze to produce hydrogen with high performance can promote the large‐scale storage of electricity energy generated from the sustainable green energy sources such as solar, wind, waterfall, geothermal, etc.^[^ ^1^, ^2^, ^3^ ^]^ Among those devices, lithium (Li)‐ion batteries are currently at the forefront of market share, research, and development. However, Li will eventually be in short supply, leading to a questionable sustainability, and the formation of Li dendrites during the battery charge/discharge can pose several safety issues.^[^ ^4^, ^5^, ^6^, ^7^, ^8^ ^]^ Therefore, there is a need to replace Li with low‐cost and safe metal anodes. Due to the unique diagonal rule of the periodic table, Mg and Li have similar chemical properties. It is worth mentioning that Mg is abundant in the earth's crust, ≈104 times more than Li. Moreover, Mg is much less active than Li, which can dramatically improve the safety manufacture and performance of the batteries.^[^ ^9^, ^10^, ^11^ ^]^ Unfortunately, there are currently the limited promising cathode materials for Mg^2+^ storage. When Mg^2+^ with high charge density are intercalated into the crystalline structure of the host material, the surrounding electrostatic field of the host lattice can be changed dramatically, leading to high diffusion energy barriers of Mg^2+^, resulting in a difficult Mg‐intercalation process, and the high volume change of cathode material during cycling processes can also leads to a rapid capacity decay.^[^ ^12^, ^13^, ^14^, ^15^ ^]^ Therefore, choosing a suitable cathode host electrode material with optimum structural design is the key to solving the problem of Mg‐ion batteries.

Based on Li/Na‐ion batteries and Li/Na‐S batteries research progress,^[^ ^16^, ^17^ ^]^ it is obvious that Li/Na‐S batteries have higher theoretical specific capacities, suggesting that the increase of non‐metallic elements in electrode materials is conducive to the increase of theoretical specific capacity, and can attribute to the formation of anions of those non‐metallic elements. These anions can provide more reversible redox active sites for embedding active metal ions.^[^ ^18^, ^19^, ^20^ ^]^ Therefore, the anion‐rich electrode materials have received much attention recently. For example, Nazar et al. reported layered anion‐rich TiS_2_ as a promising positive electrode intercalation material for Mg‐ion batteries, providing the stabilized capacity of 115 mAh g^−1^ in a Mg‐ion full cell and Jin et al. prepared VS_2_ nanoflowers with highly reversible discharge capacity (245 mAh g^−1^ at 100 mA g^−1^).^[^ ^9^, ^13^, ^15^, ^21^ ^]^ These materials showed a 2D laminar crystal structure with wide crystal plane spacing for the transport of Mg^2+^ within the crystal and provided space for the electrode deformation of the material during the insertion of Mg^2+^. These studies have not only demonstrated the obvious advantages of anion‐rich electrode materials for the storage of Mg^2+^, but also suggested the design of regular pore structures for the transportation of Mg^2+^ as a general strategy to improve the rate performance and cycle life of Mg‐ion batteries. Among anion‐rich transition metal compounds, the crystal structure of NiS_2_ possesses both the highly electronegative S—S bonds and the abundant regular 1D pore structure similar to metal–organic frameworks.^[^ ^22^, ^23^, ^24^ ^]^ The former can generate attraction with Mg^2+^ to facilitate the embedding of Mg^2+^; the latter displays a much larger pore diameter than that of Mg^2+^ and can act as the directional transport channels of Mg^2+^. Compared with the 2D layer structure, the single transport direction of Mg^2+^ can ensure more efficient transport of Mg^2+^. These features endow NiS_2_ with a great potentiality in Mg‐ion batteries. To our knowledge, there are no reports of NiS_2_ being used as a cathode material for Mg‐ion batteries.

In our research, NiS_2_ has been developed as a candidate for cathode material in Mg‐ion batteries, which has rarely been reported in the literature to our best knowledge. However, this anion‐rich NiS_2_ has poor electronic conductivity, which is demonstrated by the kinetic slowness of NiS_2_ as a conversion‐type cathode material for Li‐ion batteries.^[^ ^25^ ^]^ In general, the combination of electrode material with high conductivity carbon‐based material is considered to be an effective strategy to improve its conductivity.^[^ ^26^, ^27^, ^28^ ^]^ According to recent studies, the related composite methods can be divided into carbon coating technology and in situ growth technology on the surface of carbon material. Carbon coating technology can not only improve the electrical conductivity of electrode materials, but also limit the deformation of electrode materials due to its mechanical strength, thus improving the utilization rate and cycle life of the electrode. However, the coating of carbon on the electrode material can reduce the contact area between electrode material and the electrolyte, resulting in a large concentration polarization.^[^ ^29^, ^30^, ^31^ ^]^ Although the in situ growth technology on the surface of carbon material can improve the conductivity of the electrode material without weakening the contact area between electrolyte and the electrode material, the structural collapse caused by the structural deformation of the electrode material cannot be improved.^[^ ^32^, ^33^ ^]^ Therefore, to obtain NiS_2_ with high Mg^2+^ storage performance, it is necessary to further optimize these two composite technologies. It is well known that the irreversible structural deformation and the large difference in the ratio of internal and external deformation of electrode material are the main factors leading to the structural collapse of electrode materials. The nanoscale construction of electrode material is recognized to be effective in shortening the ionic and electronic transport paths and multiplying the active sites, thus weakening the irreversible deformation caused by the polarization phenomenon, and significantly improving the deformation uniformity of the electrode material by improving the utilization rate. Therefore, the simultaneous nanoscale construction of NiS_2_ and its in situ growth on the surface of highly conductive carbon material is expected to assemble the outstanding Mg^2+^ storage material.

In this work, of the anion‐rich NiS_2_ it is explored as the active material, which is anchored on the surface of the nickel (Ni)‐based carbon nanotubes to produce the NiS_2_/NCNTs for Mg‐ion batteries. The large cavity structure of NiS_2_ allows Mg^2+^ to shuttle freely, and the magnesiation/demagnesiation processes are found to involve the dissociation/formation of S−S bonds. The addition of NCNTs promotes charge transfer kinetics and improves the structural stability of the composite. Impressively, electrochemical characterization demonstrates a discharge capacity of 244.5 mAh g^−1^ at 50 mA g^−1^ and also excellent multiplier performance of 94.7 mAh g^−1^ at 1000 mA g^−1^. The assembled battery can be cycled more than 2000 cycles at 200 mA g^−1^. This work presents a high‐performance NiS_2_ and NiS_2_/NCNTs for Mg‐ion batteries cathodes and enables the in situ growth of NiS_2_ on NCNTs, which is important for the design of micro and nanostructures for Mg cathodes and the improvement of Mg storage performance.

## 2 Results and Discussion

**Figure 1:**
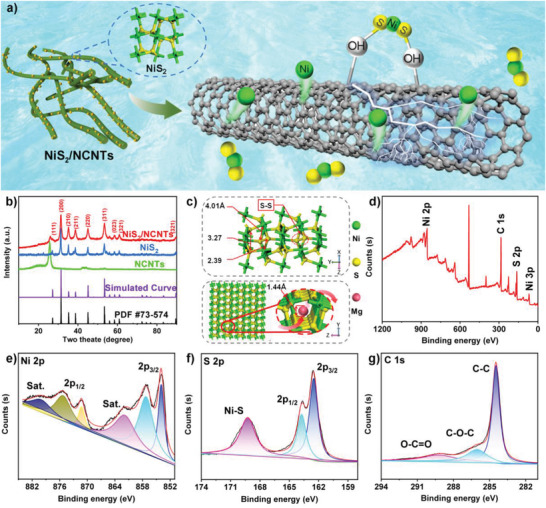
a) Diagram of NiS_2_ nanoparticles anchored on the surface of NCNTs. b) XRD pattern and c) diagram of the crystal structure of NiS_2_. d) Full XPS spectrum of NiS_2_/NCNTs, e) high‐resolution Ni 2p XPS spectrum, f) S 2p XPS spectrum, and g) C 1s XPS spectrum.

**Figure** **1**a schematically describes the in situ growth of NiS_2_ nanoparticles on NCNTs. Ni^2+^ (Figure S1, Supporting Information) and hydrophilic functional (Figures S2 and S3, Supporting Information) groups on the surface of NCNTs have a chemical affinity for S^2−^ and Ni^2+^, respectively, resulting in the preferential in situ growth of NiS_2_ crystal seeds on the surface of NCNTs.^[^ ^34^, ^35^, ^36^, ^37^, ^38^, ^39^, ^40^ ^]^ As the reaction progresses, NiS_2_ crystal seeds grow gradually, and the nanoparticles of NiS_2_ are assembled on the surface of NCNTs. The 3D NCNTs as the through–through electron transport paths can make up for the poor electronic conductivity of anion‐rich NiS_2_ electrode material. Meanwhile, the nanoscale particles of NiS_2_ can effectively shorten the transport paths of electron and Mg^2+^, thus contributing to high utilization. The XRD pattern of NCNTs, NiS_2_, and NiS_2_/NCNTs composite are shown in Figure 1b. Obviously, the XRD curve of NiS_2_/NCNTs composite contains the diffraction peaks of both NCNTs and NiS_2_, where the main diffraction peaks of NiS_2_ are consistent with the standard card (JCPDS No 73–574), and the sharp and robust diffraction peaks indicate high crystallinity. The X‐ray diffraction peak located at 27.2°, 31.5°, 35.3°, 38.8°, 45.1°, 53.3°, 58.5°, 61.0°, and 89.7°, can be attributed to the (111), (200), (210), (211), (220), (311), (023), (321), and (511) crystal planes, respectively. No heterochromatic peaks of NiO and NiS are observed in the XRD images, indicating the high purity of the synthesized NiS_2_. Because of the composition of NCNTs, a prominent diffraction peak can be observed at 26°, confirming the formation of NiS_2_/NCNTs nanocomposite.^[^ ^41^ ^]^ According to the standard card of NiS_2_, the crystal structure of NiS_2_ is shown in Figure 1c. NiS_2_ is a common cubic of pyrite structure.^[^ ^42^ ^]^ One Ni atom is connected to six S atoms. An S—S bond links two S atoms. It is noteworthy that the smallest pitch between adjacent atoms in the NiS_2_ lattice structure is measured to be 2.47 Å and the beneficial 1D cavity structure of NiS_2_ can donate the directional transmission path of Mg^2+^ (1.4 Å). Mg^2+^ can shuttle freely in the NiS_2_ crystal structure, make a possibility for NiS_2_ to store Mg^2+^. The corresponding simulated XRD pattern (Figure 1b) based on the simulated crystal structure matches all the peaks of the standard card (JCPDS No. 73–574), which confirms the correctness of the crystal structure of NiS_2_.

The X‐ray photoelectron spectroscopy (XPS) was used to analyze the surface of the prepared NiS_2_/NCNTs. As shown in Figure 1d, the survey spectrum primarily shows Ni, S, and C. The Ni 2p emission spectrum is best fitted, which is assigned to the core levels of Ni 2p_3/2_ (852–868 eV) and Ni 2p_1/2_ (870–884 eV). Two peaks at 853.8 and 871.2 eV are attributed to Ni^2+^, with two satellite peaks located at 861.9 and 880.1 eV, respectively.^[^ ^43^, ^44^ ^]^ The Ni 2p peak of NiS_2_ alone is shown in Figure S4c, Supporting Information. The peak of Ni^2+^ of NiS_2_/NCNTs is much lower than NiS_2_, possibly owing to the NCNTs entangled on the surface.^[^ ^45^ ^]^ Due to the in situ growth of NiS_2_ on NCNTs and the 3D network structure formed by the interwoven NCNTs, most of NiS_2_ is coated in the 3D network structure of NCNTs, which reduces the possibility of NiS_2_ being oxidized to a certain extent. In addition, NCNTs also contain a certain amount of nickel ions, which can also affect their diffraction peaks. Figure 1f exhibits the S 2p spectrum, two peaks present at 163.8 and 162.5 eV, corresponding to splitting the S 2p spin‐orbital (2p_1/2_, 2p_3/2_), and the peak value of 168.4 eV can be attributed to the Ni—S bond.^[^ ^46^, ^47^ ^]^ The acceptable spectrum of C 1s is shown in Figure 1g. C1s spectrum exhibits three peaks at 284.5, 286.0, and 289.2 eV, which are attributed to the C—C, C—O—C, O—C═O bonds, respectively.^[^ ^48^ ^]^ These Ni—S bonds and C—O bonds can immobilize cations (e.g., Ni^2+^, Mg^2+^). These chemical bonds could keep the whole structure stable when Mg^2+^ is detached and embedded.

**Figure 2:**
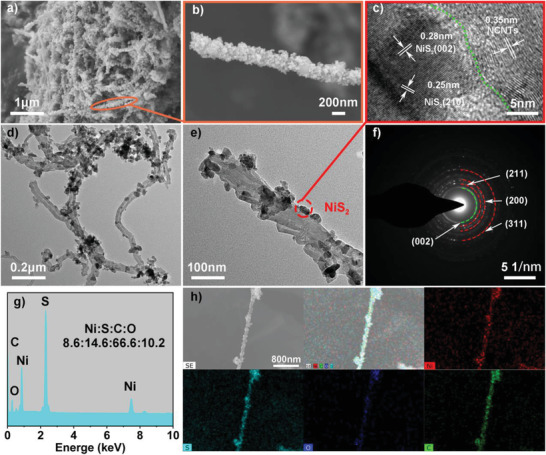
a,b) High magnification and low magnification FESEM images of NiS_2_/NCNTs, c) high resolution TEM (HRTEM) image of NiS_2_/NCNTs. d,e) High magnification and low magnification TEM images of NiS_2_/NCNTs. f) The corresponding SAED pattern of NiS_2_/NCNTs. g) EDS spectra of NiS_2_/NCNTs and h) corresponding EDS elemental mapping of Ni, S, O, and C elements.

The micromorphology of the samples was characterized by field emission scanning electron microscope (FESEM). A typical FESEM image of NiS_2_/NCNTs in **Figure** **2**a reveals an entangled network of NCNTs, and confirms the NiS_2_ nanoparticles grown in situ on NCNTs. The diameters of NiS_2_ nanoparticles are ≈20–80 nm. The large specific surface area of the nanoscale particles can provide more active sites for the electrochemical reaction.^[^ ^49^ ^]^ The cohesive network structure and fluffy structure of NCNTs also facilitate the penetration of electrolytes. Figure 2b presents the numerous small NiS_2_ nanoparticles attached to single NCNTs, and the dense arrangement of NiS_2_ nanoparticles almost covering the entire NCNTs, indicating that NCNTs and NiS_2_ nanoparticles are well composited. The microstructure of the samples was further characterized by transmission electron microscope (TEM) (Figure 2c–e). It can be seen that some of the NiS_2_ nanoparticles are detached from NCNTs due to sonication of NiS_2_/NCNTs in anhydrous ethanol solution during the sample preparation for TEM testing. So the NCNTs in the TEM images are not fully covered by the dense NiS_2_ nanoparticles as seen in the FESEM image. In addition, the high resolution transmission electron microscope (HRTEM) image in Figure 2c shows that the spacings between light and dark lattice fringes are 0.25 and 0.28 nm, which can be attributed to (210) and (200) lattice planes of NiS_2_, respectively. In addition, the lattice fringe of NCNTs can also be seen. The resolved lattice fringe with the spacing of 0.35 nm can be assigned to (002) lattice planes of NCNTs, further confirming the excellent combination of NiS_2_ and NCNTs. According to the relevant selected‐area electron diffraction (SAED) image (Figure 2f), the clearly and brightly diffraction rings with lattice feature can be attributed to the (002), (200), (210), and (211) planes. The results are consistent with those of XRD.

For the energy dispersive spectroscopy (EDS) test (Figure 2g), the atomic ratio of S:Ni is 14.6:8.6. In Figure S5f, Supporting Information, the atomic ratio of S:Ni in NiS_2_ alone is 18.9:8.5. Obviously, the atomic ratio of S and Ni in NiS_2_/NCNTs composite is lower than that in NiS_2_ due to the influence of NCNTs. Elements mapping image (Figure 2h) reveals that the NiS_2_/NCNTs is mainly composed of Ni, S, O, and C elements. As identified, NiS_2_ is uniformly distributed on surface of NCNTs, which could provide a large number of active nanoparticles for electrochemical Mg storage reactions. FESEM images of the NiS_2_ nanoparticles are shown in Figure S5a–c, Supporting Information. These images show that the nanoparticles are uniformly gathered together. The diameter of most nanoparticles was within 80 nm, which can be confirmed by TEM images (Figure S5d, Supporting Information). The individual NiS_2_ microparticles are composed of NiS_2_ nanoparticles with a similar diameter of NiS_2_ nanoparticles on the surface of NCNTs. This may be the NiS_2_ nanoparticles have a high surface energy due to their small size. In the absence of NCNTs, NiS_2_ nanoparticles are easy to agglomerate into larger microparticles. This further indicates that there is a strong interaction between NCNTs and NiS_2_, which can prevent the agglomeration of NiS_2_ nanoparticles. NCNTs can improve the dispersion of NiS_2_ and increase the contact area of NiS_2_ nanoparticles in the electrolyte, thus increasing the active sites and improving Mg storage.

**Figure 3:**
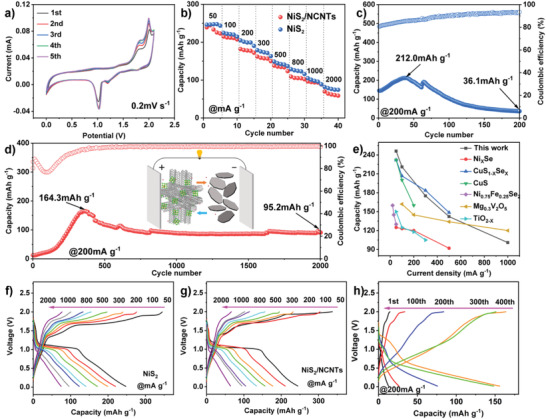
a) CV curves at 0.2 mV s^−1^of NiS_2_/NCNTs and b) rate performance of NiS_2_/NCNTs and NiS_2_. c) Long‐term cycling of NiS_2_ electrode at 200 mA g^−1^ and d) long‐term cycling of NiS_2_/NCNTs electrode at 200 mA g^−1^. e) A comparison of rate capability with previous reports. f) Charge–discharge profiles under different current densities of NiS_2,_ g) charge–discharge profiles under different current densities of NiS_2_/NCNTs, and h) charge–discharge profiles of different profiles of NiS_2_/NCNTs.

The overall electrochemical properties of the electrode materials were investigated by CV and charge–discharge tests. In **Figure** **3**a, the CV curves for the NiS_2_/NCNTs electrode show a voltage window of 0.1–2.1 V and a scan rate of 0.2 mV s^−1^. According to the test, in the initial five circles of the CV curves, all the CV curves exhibit a similar shape, indicating the high degree of reversibility of electrochemical reactions. During charging, the oxidation peak appears at 1.8 and 2.0 V, at 1.8 V, Ni is oxidized to NiS or Ni_3_S_4_, and at 2.0 V, NiS or Ni_3_S_4_ is further oxidized to NiS_2_. Due to the insertion and extraction of Mg^2+^ and the increase of cycles, the oxidation peaks are more obvious, which means the growth of reactivity and storage capacity of Mg^2+^. During the discharge process, a reduction peak appearing at 1.2 V could be attributed to the decomposition of electrolytes to form a solid‐electrolyte interface film.^[^ ^50^ ^]^ It is also that Mg is reduced MgS, a prominent reduction peak center at 1.1 V, which can be ascribed that NiS_2_ begins to transform into MgS*_X_*‐NiS_2−_ *_X_* due to the insertion of Mg^2+^. With increasing the number of cycles, the reduction peaks at 1.1 V almost overlap, signifying that the NiS_2_/NCNTs electrode has excellent reversibility and stability. The CV curves of NiS_2_ and NiS_2_/NCNTs at 0.2 mV s^−1^ scanning rate are compared, as shown in Figure S6, Supporting Information. The oxidation peak of the NiS_2_ electrode are slightly higher than those of the NiS_2_/NCNTs electrode. In Figure S7, Supporting Information, for NiS_2_/NCNTs electrode, the initial discharge capacity of the battery reaches 84.95 mAh g^−1^ at 50 mA g^−1^. However, the discharge capacity of the second cycle decreases to 20.14 mAh g^−1^ significantly, which is due to the activation of the electrode for conversion‐type materials, maybe ascribe to the irreversible trapping of Mg^2+^ too. Such an activation process is very common for conversion‐type materials.^[^ ^51^ ^]^ After 30 cycles of activation, the battery reached the maximum discharge capacity and started the rate cycles (Figure 3b). When the current density gradually increases from 50 mA g^−1^ to 100, 200, 300, 500, 1000, and 2000 mA g^−1^, the discharge capacities are 244.5, 212.5, 181.8, 158.8, 135.7, 94.7, and 69.8 mAh g^−1^ for NiS_2_/NCNTs, and the discharge capacities are 246.5, 221.0, 201.2, 175.5, 142.2, 100.9, and 78.8 mAh g^−1^ for NiS_2_, respectively. These data show that the batteries have excellent tolerance under various current densities. The results above indicate that the electrode materials containing NiS_2_ have excellent rate performance, the 1D pore structure of NiS_2_ allows Mg^2+^ to travel freely through it, and its anion‐rich properties can attract Mg^2+^ for storage. In addition, the crystal structure of NiS_2_ is stable and still has a high capacity at high current densities.

The pristine NiS_2_ and NiS_2_/NCNTs both have similar rate performance. The capacity of the NiS_2_ is slightly higher than that of the NiS_2_/NCNTs because the NCNTs do not provide the capacity contribution, which also confirms the above conjecture. The capacity of a single NCNTs electrode is almost zero at 50 mA g^−1^ current density (Figure S8, Supporting Information). The advantages of NCNTs are mainly reflected in the improvement of capacity retention and coulomb efficiency. Figure 3c illustrates the cycling performance of NiS_2_ alone at the beginning, which is the activation stage, the maximum capacity can reach 212.0 mAh g^−1^ at 200 mA g^−1^. However, after only 200 cycles, the capacity decays to 36.1 mAh g^−1^, and at the capacity retention rate of just 17%. The single NiS_2_ electrode material decays too fast during the cycling, and the coulomb efficiency is only ≈90%. It may be because the electrode materials are agglomerated (Figure S5a, Supporting Information), which could lead to the low accessibility of the electrolyte. However, the cycling performance of NiS_2_/NCNTs electrode at 200 mA g^−1^ current density (Figure 3d) presents excellent cycle stability. After 2000 cycles, the reversible capacity of the NiS_2_/NCNTs electrode still remains at 95.2 mAh g^−1^, and the capacity retention is up to 58%. At the beginning of the cycle, the capacity of the NiS_2_/NCNTs electrode gradually increases with increasing the number of cycles. After 350 cycles, the maximum capacity reaches 164.3 mAh g^−1^. The process of the capacity increase may be attributed to the activation of electrode material. Besides, as the side reaction between electrode material and the electrolyte is eliminated, the gradual exposure of the active site of the electrochemical reaction leads to the gradual improvement of the chemical reaction.^[^ ^52^ ^]^ After 600 cycles, the capacity increases due to the increased activation depth of the NiS_2_ nanoparticle electrode. Besides, the coulomb efficiency of the NiS_2_/NCNTs electrode is close to 100% with increasing the number of cycles. At the beginning of the cycles, the low coulomb efficiency can be attributed to the difficulty of Mg^2+^ intercalation. With increasing the number of cycles, the electrode material is gradually activated, which then improves the coulomb efficiency. The excellent electrical conductivity of NCNTs can facilitate the transport of electrons, the charge loss during the transport of Mg^2+^ and thus improving the coulomb efficiency. In conclusion, the addition of NCNTs can enhance the dispersion of NiS_2_ nanoparticles uniformly on the surface of the NCNTs, thus increasing the active sites of the frontal NiS_2_ nanoparticles, which is more conducive to contact with the electrolyte. Besides, the network structure of NCNTs has affluent buffer areas, which can be used as a local memory to save the electrolyte. Furthermore, NCNTs can reduce the volume change of electrode materials during the cycling process and accelerate the electrochemical reaction kinetics.^[^ ^52^, ^53^, ^54^, ^55^, ^56^, ^57^ ^]^ Figure 3e shows the rate performance of the reported cathode materials under different current densities. NiS_2_ has an excellent multiplicative property compared to other materials, especially at low current densities. Figure 3f,g depicts the corresponding charge–discharge profiles of NiS_2_/NCNTs and NiS_2_ electrodes under different current densities ranging from 50 to 1000 mA g^−1^. At the discharge platform of ≈1.1 V, the obvious charging and discharging profiles can be seen from the figure. The discharge platform is still visible at a high current rate of 2000 mA g^−1^, despite of showing a slight decline of the platform voltage. These results demonstrate that NiS_2_/NCNTs and NiS_2_ electrodes have excellent rate performance, and the polarization increases with changing current densities. The charge–discharge profiles of NiS_2_/NCNTs electrode at 200 mA g^−1^ current density are shown in Figure 3h. The charge–discharge voltage profiles of the first cycle exhibit relatively low capacity. As the reaction proceeds, the capacity of NiS_2_/NCNTs electrode reaches the maximum, then decreases gradually, and finally tends to be stable. The conclusion is reflected in the data of long cycling.

**Figure 4:**
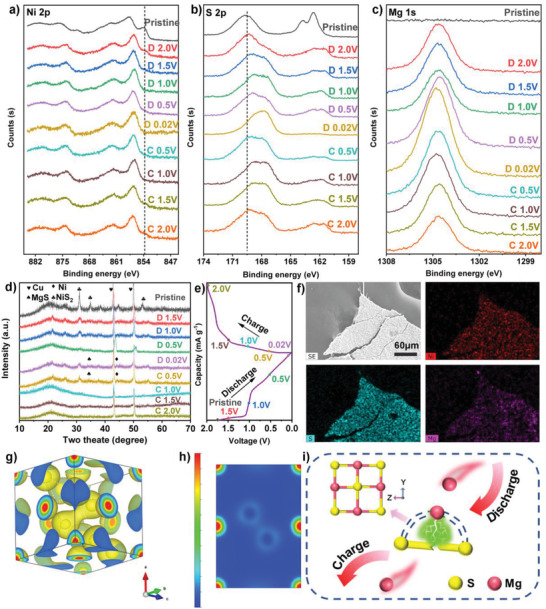
Ex situ XPS patterns at different under different charging and discharging conditions, a) XPS spectra of Ni 2p, b) XPS spectra of S 2p, and c) Mg 1s. d) Ex situ XRD patterns of the NiS_2_/NCNTs microspheres under different electrochemical stages and e) corresponding charge–discharge profiles. f) Corresponding EDS elemental mapping of Ni, S, and Mg in the electrode sheet after cycling. g) 3D charge density map and h) 2D charge density map. i) Reaction mechanism during discharge.

Ex situ XPS was employed to study the mechanism of the electrode reaction. **Figure** **4**a–c exhibits the XPS spectra at different discharge states. The storage mechanism of Mg can be explained by XPS results. Figure 4a shows the XPS peaks of Ni 2p in charges and discharge states. In the initial state, the peak at 853.7 eV corresponds to Ni^2+^. In the discharge process, the peak of Ni 2p disappears gradually, indicating that a reduction reaction takes place, and Ni^2+^ may be reduced to elemental Ni. In continuous charging, the peak of Ni^2+^ appears, suggesting the mainly reversible conversion reactions. In Figure 4b, the peaks of S 2p are composed of Ni—S bond and two sets of doublet peaks (S 2p_3/2_ and S 2p_1/2_). The peak of the Ni—S bond is 169.7 eV when pristine. During the discharge process from pristine to 0.02 V, the Ni—S bond shifts to the low binding energies and shifts back to the initial value with charging to 2.0 V. The reversible shift in binding energy indicates that the anion S is involved in redox reaction. One peak located at 162.7 eV corresponds to 2p_3/2_, while the other peak at 161.6 eV corresponds to 2p_1/2_.^[^ ^20^, ^58^ ^]^ During the discharge process, two peaks of 2p_1/2_ and 2p_3/2_ gradually diminish, and at 0.02 V in the discharge state, the two peaks are almost zero, indicating that the peak value decreases due to the attraction of S^2−^ during Mg^2+^ insertion.^[^ ^59^ ^]^ As charging proceeds, the two peaks rise slowly with charging voltage, showing excellent reversibility. At the same time, Mg 1s peak enhances/fades upon discharge/charge (Figure 4c), indicating good reversibility of the magnesiation/demagnesiation processes.^[^ ^60^ ^]^

Ex situ XRD was also used to study the electrode reaction mechanism. Figure 4d exhibits the XRD patterns of NiS_2_/NCNTs at different charging and discharging points after five cycles. The peak at 26° is attributed to NCNTs, and 42.8° and 49.9° are attributed to the copper foil as a collector. The peaks at 34.1°, 34.9°, and 53.3° are attributed to the pristine NiS_2_ electrode. The diffraction peak of NiS_2_ becomes weakened during the discharge to 0.02 V, suggesting that NiS_2_ is involved in the electrode reaction. In contrast, two peaks appear at 44.2° and 55.1°, which can be attributed to elemental Ni, and the other peaks may overlap with those of NiS_2_. When fully discharged to 0.02 V, there was a weak peak of MgS at 34.4°.^[^ ^52^ ^]^ The peak value is not apparent, which can be attributed to the low crystallinity. MgS and metal Ni can form nanoparticles, which makes it difficult to identify wide XRD peaks. During the charging process, these two peaks disappear gradually, suggesting that the electrode process may involve breaking S—S bonds and forming MgS during the discharge process, which is consistent with our conjecture. Through the ex situ XRD test, the chemical reaction equation can be deduced as follows:

(1) $$\mathrm{Ni}S_{2}+2\mathrm{Mg}↔2\mathrm{MgS}+\mathrm{Ni}$$

Figure 4f shows an image of the element mapping, where the three elements Ni, S, and Mg are uniformly distributed on the electrode sheet after 50 cycles, demonstrating the presence of the element Mg. In Figure 4g, the charge density analysis shows that in the NiS_2_ crystal structure, the negative charge is mainly concentrated near the S—S bonds. When Mg^2+^ with a positive divalent enters the NiS_2_ crystal structure, it will first adsorb near the S—S bonds where the negative charge is concentrated. Then as the S—S bonds break, a large amount of the rich anion S^2−^ is generated, and Mg^2+^ forms a weakly coordinated Mg—S bond with S^2−^ to produce MgS.^[^ ^61^, ^62^, ^63^ ^]^ In Figure 4i, based on the above findings, the reaction mechanism of the discharge process in an all phenyl complex/tetrahydrofuran solution (APC) electrolyte system with tetrahydrofuran as solvent is postulated. Due to the sufficiently large pore size in the NiS_2_ crystal structure during the discharge process, Mg^2+^ can pass freely through it. Under mild conditions, THF can promote the breakage of the S—S bonds in NiS_2_.^[^ ^20^ ^]^ The positively valent Mg^2+^ is attracted by the negatively valent S^2−^ to produce MgS, because the spacing between the S—S bonds is only 2.07 Å, Mg^2+^ is not easily inserted between the S—S bonds, which can result in weaker Mg—S bonds and easier detachment of Mg^2+^ during the charging process to complete the discharge–charge cycle.^[^ ^13^ ^]^

**Figure 5:**
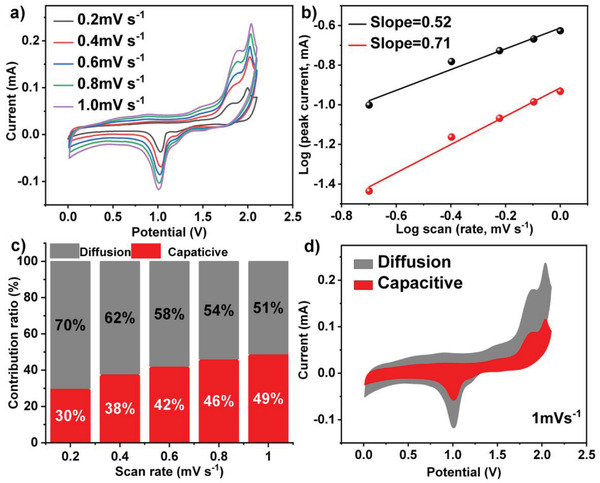
a) CV curves at different scan rates of NiS_2_/NCNTs. b) *log i‐log v* curves of NiS_2_/NCNTs. c) Capacitive and diffusion contribution in CV and d) contribution ratio at various scan rates of NiS_2_/NCNTs.

The CV curves with different scan rates of 0.2, 0.4, 0.6, 0.8, and 1.0 mV s^−1^ were measured to explore NiS_2_/NCNTs for Mg^2+^ storage. From CV curves (**Figure** **5**a) of different sweep speeds, the shapes are well preserved with increasing scan rate from 0.2 to 1.0 mV s^−1^. The reduction peak and oxidation peak at 1.1 and 1.8 V, respectively, have similar reduction and oxidation peaks in each curve with different scan rates. The relationship between the current (*i*) and the scan rate (*ν*) measured in the CV curve allows a qualitative analysis of the extent of the capacitive effect:

(2) $$i=av^{b}$$

(3) logi=loga+b*logν

Where a and b both are constant. The value of b is between 0.5 and 1.0. The b value of 0.5 indicates a total diffusion‐controlled behavior, whereas 1.0 represents a capacitive process.^[^ ^64^, ^65^ ^]^ They can be determined from the slope of the *log i* versus *log ν* plot. The slopes of *log ν*–*log i* plots are 0.25 and 0.71, respectively. Besides, the peak current gradually enhances with increasing the scan rate. The results reveal that the storage mechanism of Mg^2+^ has excellent non‐diffusion limits because of NCNTs covered by NiS_2_ nanoparticles. As seen from the FESEM images, this unique structure exposes many active sites for charge storage, suggesting that the non‐diffusion limit current in the NiS_2_/NCNTs electrode is important. By using the formula, the capacitive contribution ratio can be ascribed to the equation:

(4) $$i\left(V\right)=k_{1}\nu +k_{2}\nu^{1/2}$$

Where the contribution from pseudo‐charge storage is *k_1_v*, and the insertion type capacity is *k_2_ν^1/2^*.^[^ ^66^, ^67^, ^68^, ^69^ ^]^ The non‐diffusion limiting current is obtained by calculating the *k_1_v* value of the red region in Figure 5c. When the sweep rate are 0.2, 0.4, 0.6, 0.8, and 1.0 mV s^−1^, the capacitance contribution rate is 30%, 38%, 42%, 46%, and 49%, respectively. The quantified results (Figure 5d) show that the capacitive capacity is improved gradually with increasing the scan rate and finally reaches a maximum value of 49% at 1.0 mV s^−1^, indicating the enhanced pseudo‐capacitive behavior of nanoparticles morphology. Further Nyquist plots obtained by electrochemical impedance spectroscopy (EIS) measurements (Figure S9, Supporting Information) show that NiS_2_/NCNTs has a lower resistance than pristine NiS_2_, indicating a faster charge transfer rate, which is due to the excellent conductivity of NCNTs.

## 3 Conclusions

In summary, inspired by anion‐rich NiS_2_, NiS_2_/NCNTs composite is prepared by in situ growth. The large cavity crystal structure of NiS_2_ allows the passage of Mg^2+^, and the anion‐rich nature promotes redox reactions. The processes of magnesiation/demagnesiation involve the dissociation/formation of S—S bonds. As the first proposed cathode material, NiS_2_/NCNTs shows the better specific capacity and excellent ratio performance, giving a high capacity of 244.5 mAh g^−1^ at 50 mA g^−1^, an outstanding rate performance (94.7 mAh g^−1^ at 1000 mA g^−1^). Benefited from the electrical conductivity of NCNTs and the fact that NCNTs reduce the volume change of the material during cycling, NiS_2_/NCNTs exhibits excellent long cycle performance (58% capacity retention after 2000 cycles). This research explores new Mg storage materials and offers a promising option for developing cathode materials for Mg‐ion batteries.

## 4 Experimental Section

### Synthesis of NiS_2_ Nanoparticles

The NiS_2_ sample was synthesized using a typical solvothermal method. Nickel nitrate hexahydrate (1.5 mmol, 0.4362 g, Ni(NO_3_)_2_·6H_2_O), sodium thiosulfate (1 g, Na_2_S_2_O_3_), and absolute alcohol (16 mL, C_2_H_5_OH) were added in 30 mL Teflon‐lined autoclave under magnetic stirring until all the solids were dissolved completely. The autoclave was heated for 36 h at 150 °C, then this autoclave was cooled down to room temperature naturally, to obtain a black precipitation, which was then separated by centrifugation and washed six to seven times with distilled water and absolute ethanol, dried overnight in a vacuum oven at 80 °C, to obtain the target NiS_2_ particle sample.

### Synthesis of NiS_2_/NCNTs Composites

For synthesis of NiS_2_/NCNTs, Ni‐Based carbon nanotubes (NCNTs) (30 mg), nickel nitrate hexahydrate (1.5 mmol, 0.4362 g, Ni(NO_3_)_2_·6H_2_O), sodium thiosulfate (1 g, Na_2_S_2_O_3_), and absolute alcohol (16 mL, C_2_H_5_OH) were added in 30 mL Teflon‐lined autoclave under magnetic stirring until all the solids were dissolved completely. The followed procedure for heat‐treatment and collection of NiS_2_/NCNTs sample was the same as that of the NiS_2_ sample described above.

### Materials Characterization

The morphology and elemental composition of the samples were analyzed by FESEM (Merlin Compact, ZEISS). The nanostructures of the resulting samples were recorded using a TEM (TecnaiG2 F20, FEI). The composition of elements was analyzed by energy dispersive spectroscopy (EDS) under a 20 V electron beam. The crystal structure of the as‐prepared materials was recorded on X‐ray diffraction (XRD, Ultima IV, Rigaku) with Cu‐K*α* radiation from 10° to 90° at a scanning rate of 10° min^−1^. Fourier‐transform infrared (FTIR) spectra of the nanostructures were recorded with a Thermo Fisher Nicolet iS50 FTIR, which had a scan range of 4000 to 400 cm^−1^. XPS was performed using an X‐ray photoelectron spectroscopy (K‐alpha, Thermo Fisher) with Al K*α* X‐rays as the excitation source.

### Electrochemical Measurements

The cathodic working electrodes were fabricated by coating NiS_2_ or NiS_2_/NCNTs with acetylene black and polyvinylidene fluoride (PVDF) binder at a ratio of 8:1:1 on copper foil collector. Coin‐type battery cells (CR2032) were assembled in an Ar‐filled glove box with water and oxygen contents below 0.1 ppm. The cells contained Mg foil anode polished by SiC sandpaper and cleaned before use. All phenyl complex/tetrahydrofuran solution (APC) as the electrolyte and glass microfiber filter (Whatman, Grade GF/D) as the separator. Electrochemical measurements (cyclic voltammetry [CV] and electrochemical impedance spectroscopy [EIS]) were performed on the electrochemical workstation (brilliance chi660e). The galvanostatic discharge–charge performances were recorded by the XinWei multichannel battery test system using the voltage range of 0.1–2.1 V.

### Computational Methods

The spin‐unrestricted density functional theory (DFT) calculations were performed by the Vienna Ab initio Simulation Package (VASP) with the GGA‐PBE exchange–correction functional. The E‐cut was set to be 400 eV, and the 5 × 5 × 5 Monkhorst‐Pack k‐point grid was adopted to sample the Brillouin zone. The convergence criteria for energy and force were set to be 10^−5^ eV and 0.02 eV Å^−1^, respectively.

## Conflict of Interest

The authors declare no conflict of interest.

## Supporting information

Supporting information

## Data Availability

Research data are not shared.
