# Supplementary material for: In Situ Anchoring Anion‐Rich and Multi‐Cavity NiS2 Nanoparticles on NCNTs for Advanced Magnesium‐Ion Batteries
Source: Adv Sci (Weinh). 2022 Apr 24;9(18):2200067. doi: 10.1002/advs.202200067 (PMC9218762; doi:10.1002/advs.202200067)
Supplement: Supplementary file 1 — Supporting information [file ADVS-9-2200067-s001.pdf]

ADVANCED SCIENCE
Open Access

Supporting Information

for *Adv. Sci.*, DOI 10.1002/advs.202200067

In Situ Anchoring Anion-Rich and Multi-Cavity NiS_2 Nanoparticles on NCNTs for Advanced Magnesium-Ion Batteries

Zisen Ye, Ping Li, Wutao Wei, Chao Huang, Liwei Mi*, Jinglai Zhang and Jiujun Zhang**

Supporting Information

In-situ anchoring anion-rich and multi-cavity NiS_2 nanoparticles on NCNTs for advanced magnesium-ion batteries

Zisen Ye, Ping Li, Wutao Wei, Chao Huang, Liwei Mi* Jinglai Zhang and Jiujun Zhang**

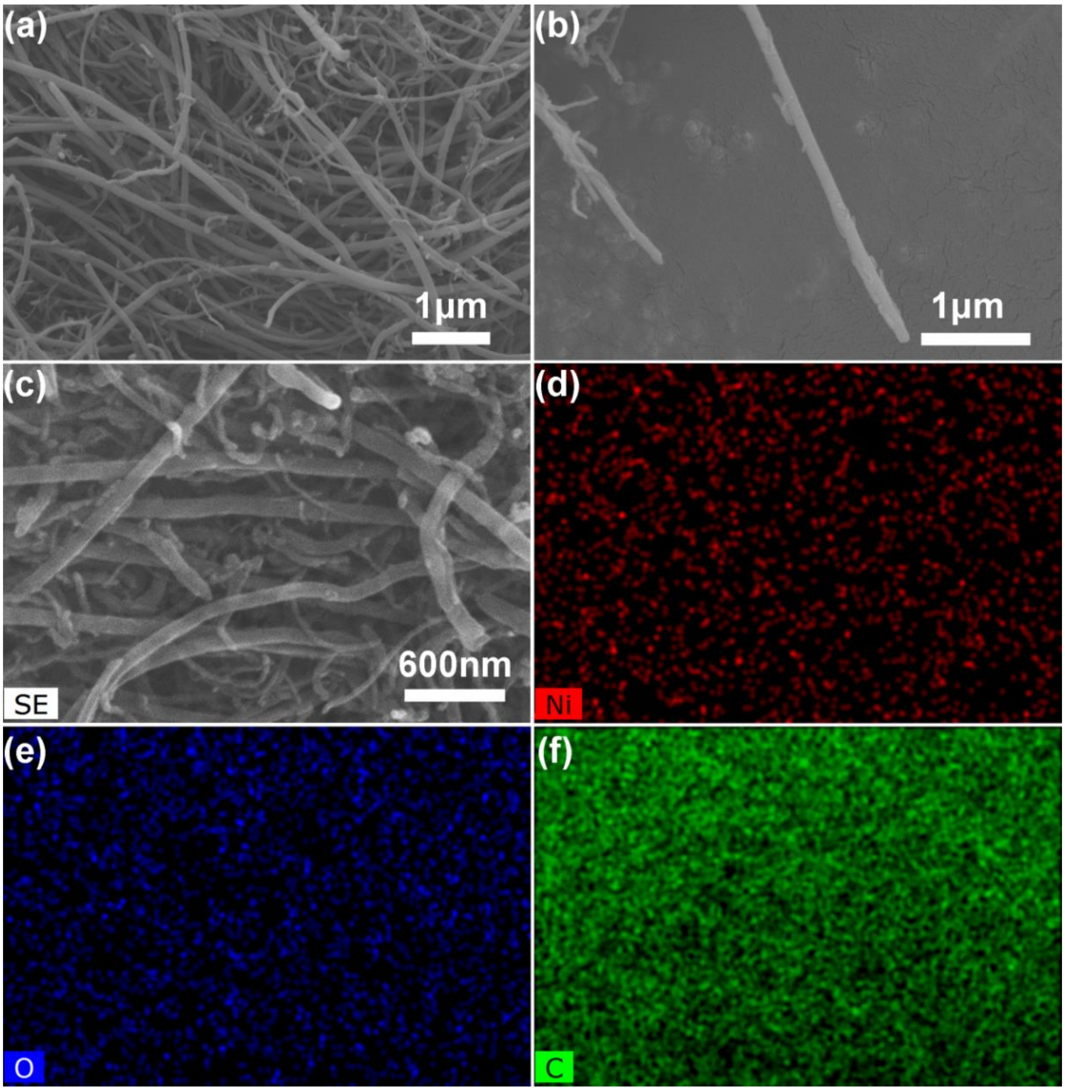

Figure S1. (a-b) High magnification and low magnification FESEM images of NCNTs, (c-f) corresponding EDS elemental mapping of Ni, O and C elements.

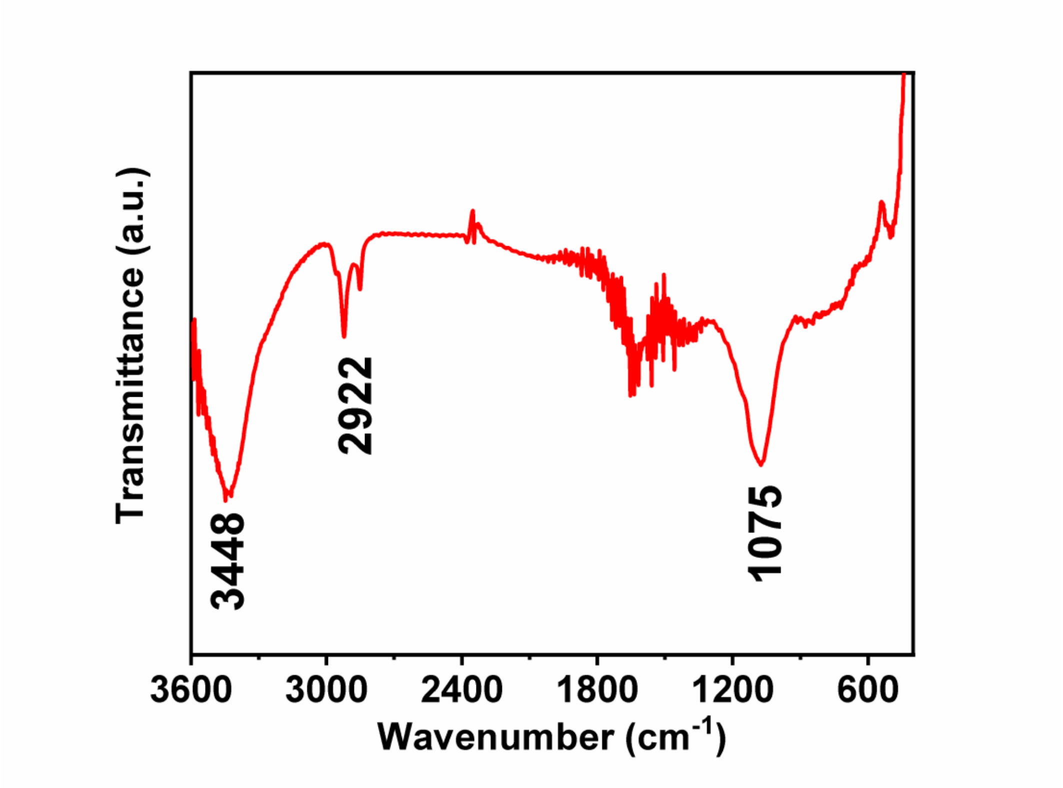

Figure S2. FTIR spectra of NCNTs .

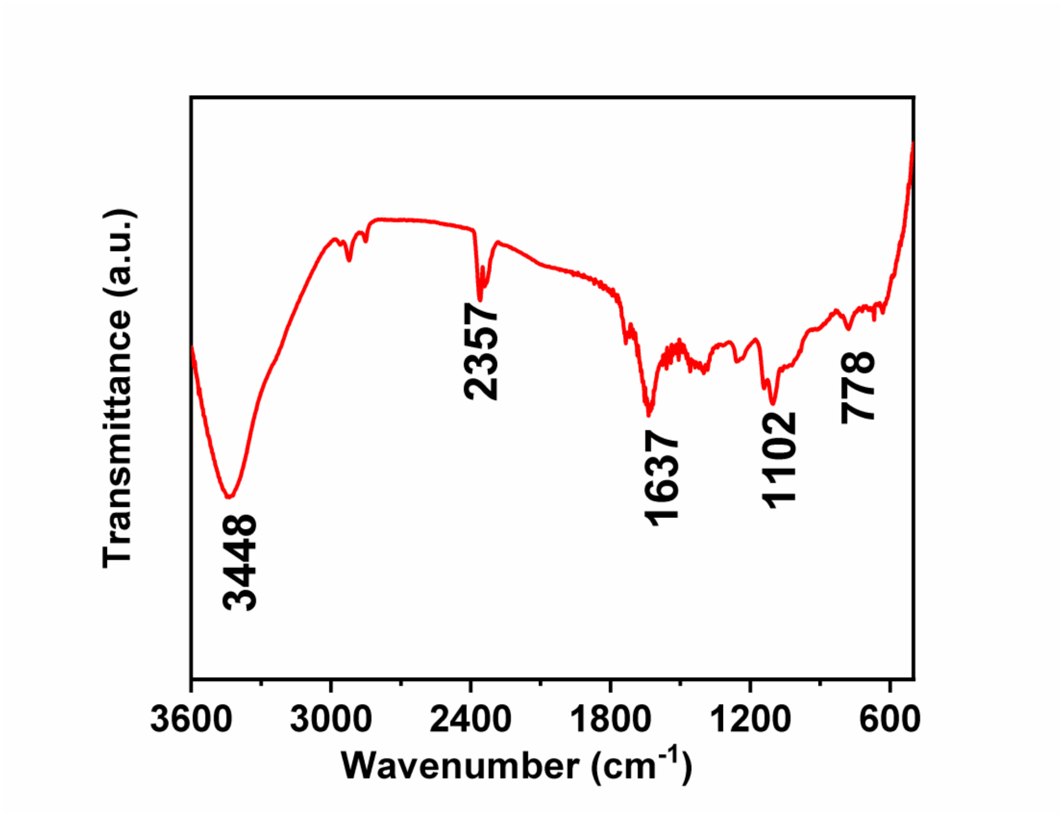

Figure S3. FTIR spectra of NiS_2.

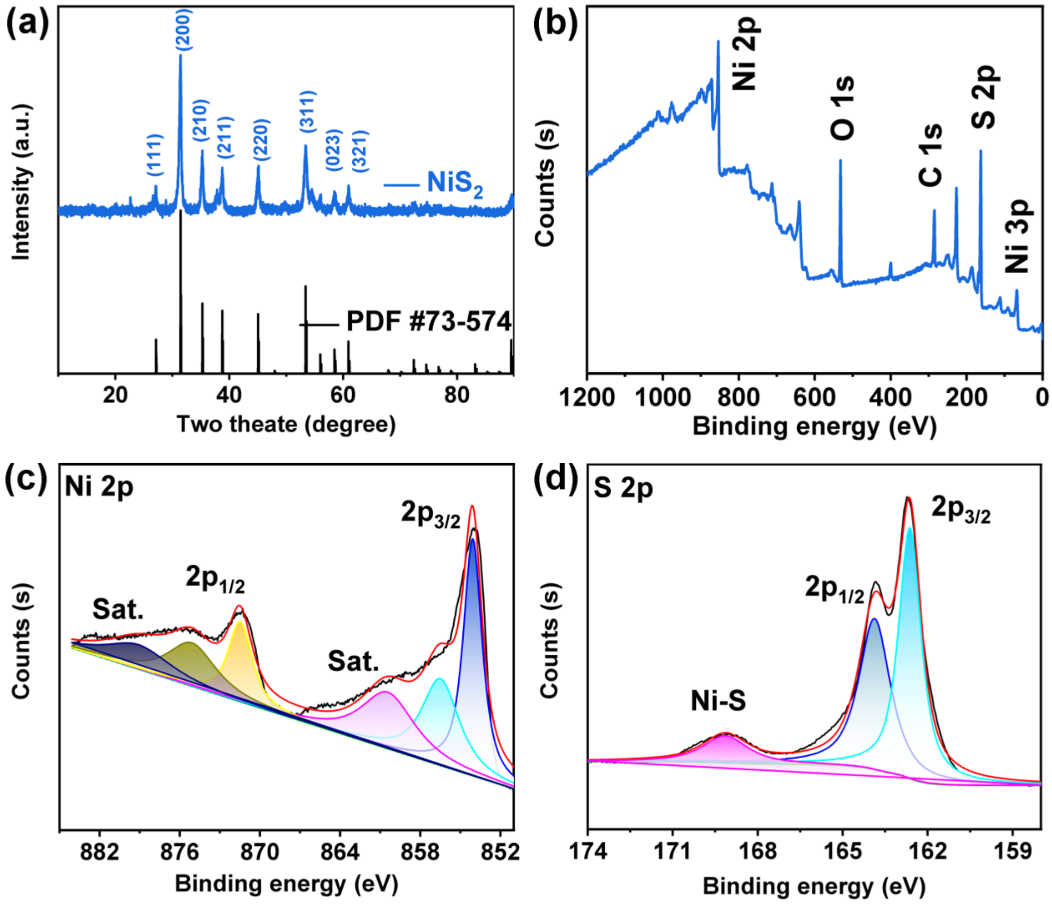

Figure S4. (a) XRD pattern of NiS_2, (b) full XPS spectrum of NiS_2, (b) high-resolution Ni 2p XPS spectrum, (e) S 2p XPS spectrum.

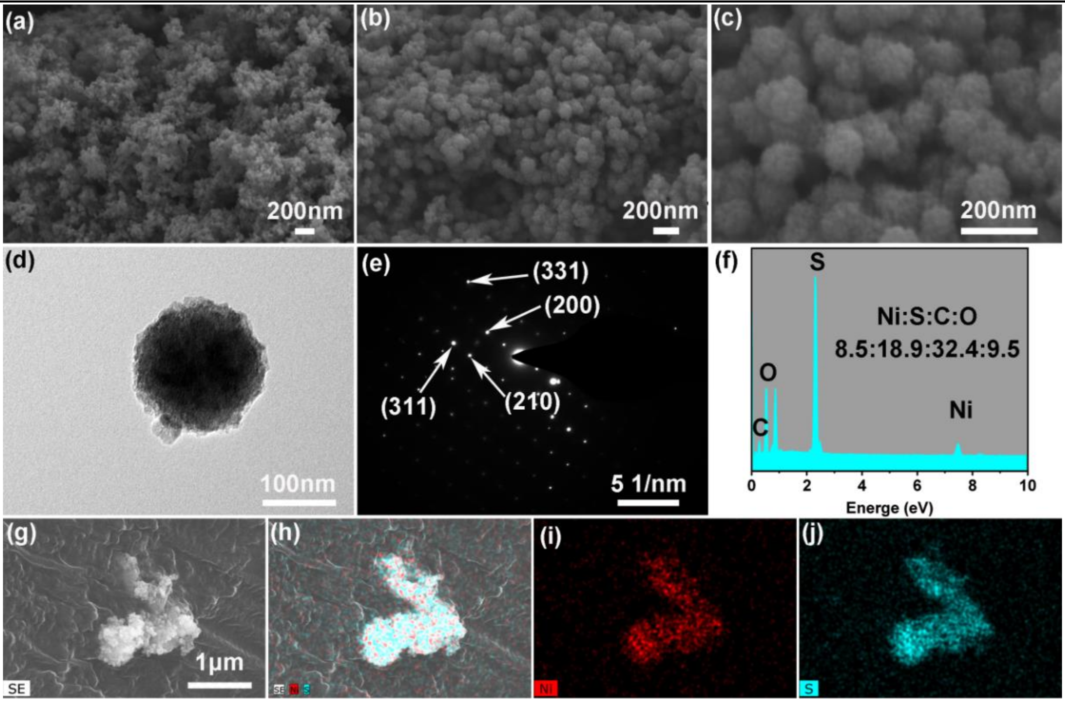

Figure S5. (a-c) High magnification and low magnification FESEM images of NiS_2, (d) TEM image of NiS_2, (e) SAED pattern of NiS_2, (g) EDS spectra of NiS_2, and (h-m) corresponding EDS elemental mapping of Ni, S, O and C elements.

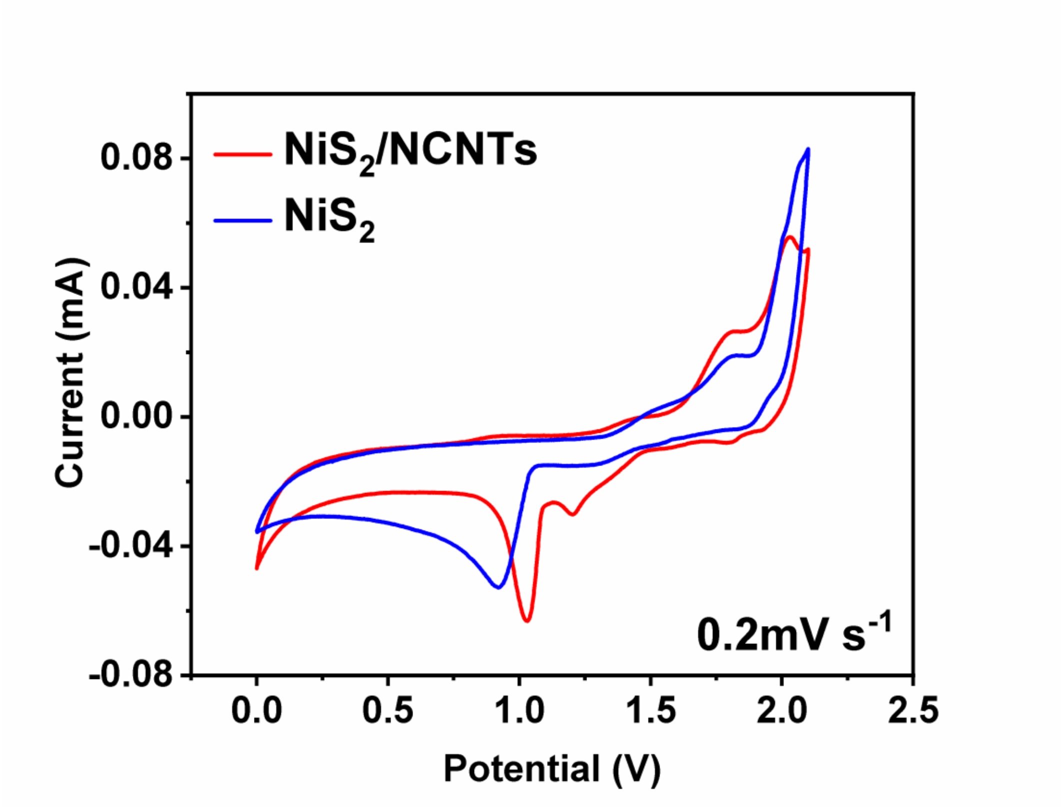

Figure S6. Comparison of CV curves of NiS_2 and NiS_2/NCNTs at 0.2mV s^{-1}.

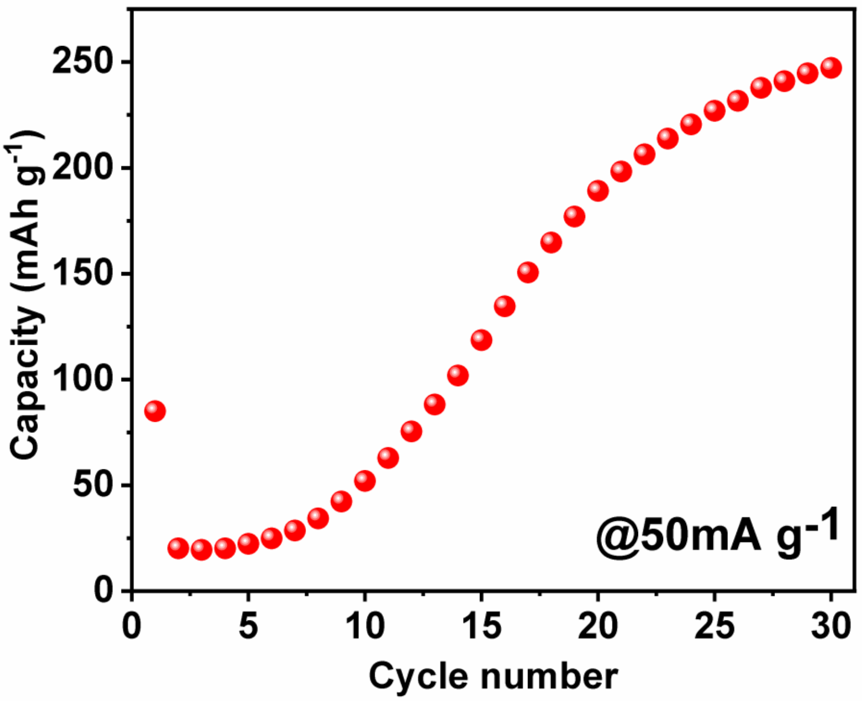

Figure S7. The electrode activation process of NiS_2/NCNTs.

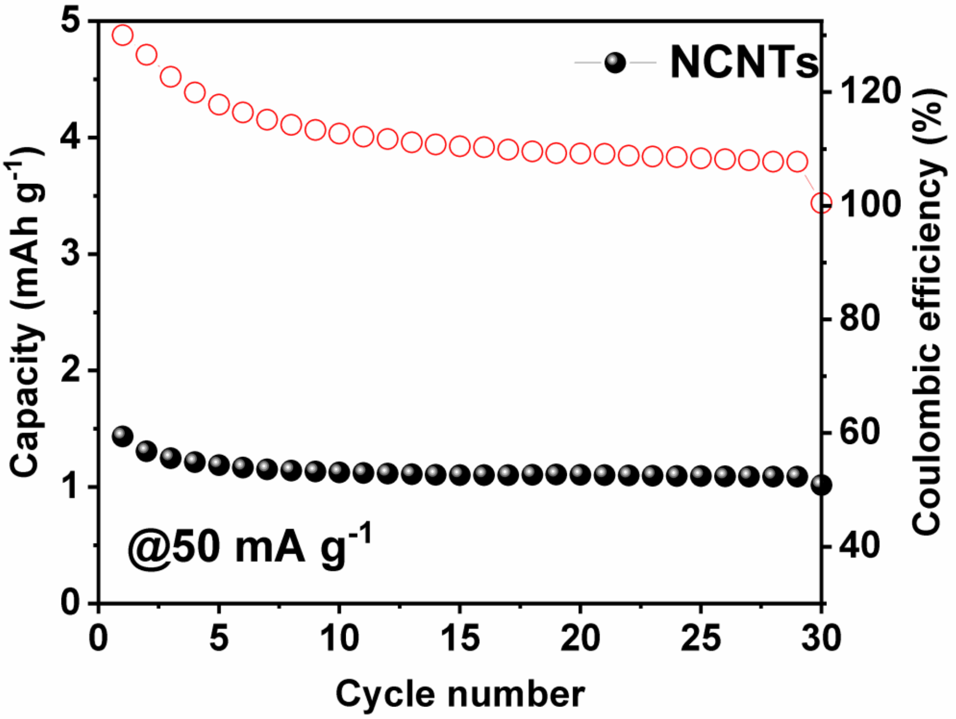

Figure S8. The cycling of NCNTs.

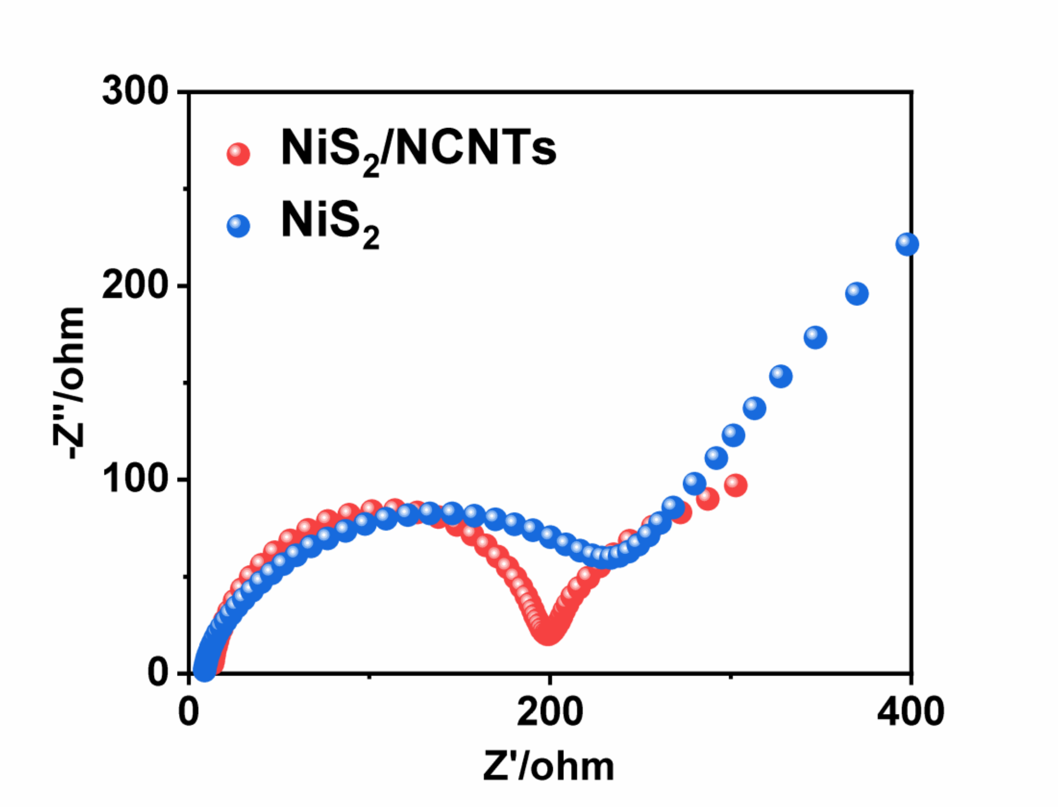

Figure S9. Nyquist plots of NiS_2 and NiS_2/NCNTs at pristine.

Table S1. Summary about the Mg storage properties of some related electrode materials.

Cathode Material	Voltage window (V vs. Mg/Mg^{2+})	Discharge capacity (mAh g^{-1})	Cycle numbers	Ref
MoS_2	0.0-3.0	116 @ 20	50	[1]
Mo_6S_8	0.5-1.8	110 @ 100	500	[2]
CuS(50°C)	0.0-2.0	148 @ 20	30	[3]
CuS	0.01-2.4	150 @ 500	1000	[4]
CuS	0.01-2.0	255 @ 20	200	[5]
CuS/CNTs	0.2-1.8	165 @ 100	100	[6]
CuS	0.3-2.2	230 @ 50	350	[7]
$CuS_{1-x}Se_X$	0-2.0	232 @ 50	90	[8]
CoS	0.1-2.0	125 @ 50	30	[9]
CoS	0-1.8	376 @ 20	80	[10]
VS_2	0.2-2.2	212 @ 200	600	[11]
VS_4	0.2-2.2	95 @ 10	400	[12]
VS_4	0.2-2.2	137 @ 50	800	[13]
Ni_3Se_4	0.01-2.0	106 @ 50	100	[14]
$Ni_{0.85}Se$	0.02-2.5	92 @ 500	500	[15]
$Ni_{0.75}Fe_{0.25}Se_2$	0.02-2.0	190 @ 10	500	[16]
NiS_2/NCNTs	0.02-2.0	245 @ 50	2000	This work

References

[1] Y. Liu, L. Fan, L. Jiao, *J. Power Sources*. **2017**, *340*, 104.

[2] M. Mao, Z. Lin, Y. Tong, J. Yue, C. Zhao, J. Lu, Q. Zhang, L. Gu, L. Suo, Y. Hu, H. Li, X. Huang, L. Chen, *ACS Nano*. **2020**, *14*, 1102.

[3] F. Xiong, Y. Fan, S. Tan, L. Zhou, Y. Xu, C. Pei, Q. An, L. Mai, *Nano Energy*. **2018**, *47*, 210.

[4] Y. Shen, Y. Wang, Y. Miao, M. Yang, X. Zhao, X. Shen, *Adv. Mater*. **2020**, *32*, 1905524.

[5] Z. Wang, S. Rafai, C. Qiao, J. Jia, Y. Zhu, X. Ma, C. Cao, *ACS Appl. Mater. Interfaces*. **2019**, *11*, 7046.

[6] Y. Zhang, Y. Li, Y. Wang, R. Guo, W. Liu, H. Pei, G. Yin, D. Ye, S. Yu, J. Xie, *J. Colloid. Interf. Sci*. **2019**, *553*, 239.

[7] M. Wu, Y. Zhang, T. Li, Z. Chen, S. Cao, F. Xu, *Nanoscale*. **2018**, *10*, 12526.

[8] Z. Wang, Y. Zhu, C. Qiao, S. Yang, J. Jia, S. Rafai, X. Ma, S. Wu, F. Ji, C. Cao, *Small*. **2019**, *15*, 1902797.

[9] D. He, D. Wu, J. Gao, X. Wu, X. Zeng, W. Ding, *J. Power Sources*. **2015**, *294*, 643.

[10] M. Pan, J. Zou, R. Laine, D. Khan, R. Guo, X. Zeng, W. Ding, *J. Mater. Chem. A*. **2019**, *7*, 18880.

[11] X. Xue, R. Chen, C. Yan, P. Zhao, Y. Hu, W. Kong, H. Lin, L. Wang, Z. Jin, *Adv. Energy. Mater*. **2019**, *9*, 1900145.

[12] Z. Li, S. Ding, J. Yin, M. Zhang, C. Sun, A. Meng, *J. Power Sources*. **2020**, *451*, 227815.

[13] Y. Wang, Z. Liu, C. Wang, X. Yi, R. Chen, L. Ma, Y. Hu, G. Zhu, T. Chen, Z. Tie, J. Ma, J. Liu, Z. Jin, *Adv. Mater*. **2018**, *30*, 1802563.

[14] L. Wei, R. Lian, Y. Zhao, Y. Meng, L. He, Y. Yu, G. Chen, Y. Wei, *ACS Appl. Mater. Interfaces*. **2020**, *12*, 9316.

[15] D. Chen, J. Shen, X. Li, S. Cao, T. Li, W. Luo, F. Xu, *J. Energy Chem*. **2020**, *48*, 226.

[16] L. Zhou, F. Xiong, S. Tan, Q. An, Z. Wang, W. Yang, Z. Tao, Y. Yao, J. Chen, L. Mai, *Nano Energy*. **2018**, *54*, 360.
